# Transition to Metallic and Superconducting States Induced by Thermal or Electrical Deoxidation of the Dislocation Network in the Surface Region of SrTiO_3_

**DOI:** 10.3390/nano14231944

**Published:** 2024-12-04

**Authors:** Krzysztof Szot, Christian Rodenbücher, Krzysztof Rogacki, Gustav Bihlmayer, Wolfgang Speier, Krystian Roleder, Franciszek Krok, Hugo Keller, Arndt Simon, Annette Bussmann-Holder

**Affiliations:** 1 A. Chełkowski Institute of Physics, University of Silesia, 41-500 Chorzów, Poland; krzysztof.szot@us.edu.pl (K.S.); krystian.roleder@us.edu.pl (K.R.); 2 Institute of Energy Technologies (IET-4), Forschungszentrum Jülich GmbH, 52425 Jülich, Germany; 3 Institute of Low Temperature and Structure Research, Polish Academy of Sciences (PAS), 50-050 Wrocław, Poland; k.rogacki@intibs.pl; 4 Peter Grünberg Institute (PGI-1) and JARA-FIT, Forschungszentrum Jülich GmbH, 52425 Jülich, Germany; g.bihlmayer@fz-juelich.de; 5 Peter Grünberg Institute (PGI-SO) and JARA-FIT, Forschungszentrum Jülich GmbH, 52425 Jülich, Germany; w.speier@fz-juelich.de; 6 M. Smoluchowski Institute of Physics, Jagiellonian University, 30-348 Kraków, Poland; franciszek.krok@uj.edu.pl; 7 Physik-Institute of the University of Zürich, University of Zürich, 8057 Zürich, Switzerland; keller@physik.uzh.ch; 8 Max-Planck-Institute for Solid State Research, 70569 Stuttgart, Germany; a.simon@fkf.mpg.de (A.S.); a.bussmann-holder@fkf.mpg.de (A.B.-H.)

**Keywords:** superconductivity, thermal reduction, dislocations

## Abstract

The question as to why deoxidized SrTiO_3−δ_ becomes metallic and superconducting at extremely low levels of oxygen vacancy concentration has been a mystery for many decades. Here, we show that the real amount of effused oxygen during thermal reduction, which is needed to induce superconducting properties, is in the range of only 10^14^/cm^3^ and thus even lower than the critical carrier concentrations assumed previously (10^17^–10^19^/cm^3^). By performing detailed investigations of the optical and electrical properties down to the nanoscale, we reveal that filaments are forming during reduction along a network of dislocations in the surface layer. Hence, a reduced epi-polished SrTiO_3−δ_ crystal has to be regarded as a nano-composite consisting of a perfect dielectric matrix with negligible carrier density, which is short-circuited by metallic filaments with a local carrier density in the range of 10^20^/cm^3^. We present that electro-degradation leads to a more pronounced evolution of filamentary bundles and thus can generate a superconducting state with higher *T*_C_ than thermal reduction. These findings indicate that traditional homogeneous models of superconductivity in self-doped SrTiO_3−δ_ need to be revised, and we propose an alternative explanation taking into account the coexistence of metallic dislocation cores with polar insulating regions allowing for polaronic coupling.

## 1. Introduction

Since the discovery that the reduction of strontium titanate (STO) at high temperatures and low oxygen activity (established by vacuum, reducing atmosphere, or oxygen getters) can induce metallic or superconducting properties six decades ago [1,2,3,4,5,6,7], the reported minimal concentration for the doping level, which is necessary for the mentioned transition, has been permanently shifted towards lower levels. Recently reported critical concentrations for metallicity are in the range of 10^15^ charge carriers per cm^3^ [8], which is three orders of magnitude smaller than that predicted by the classical Mott criterion for STO [9]. Furthermore, superconductivity in STO has been induced by (nominally) homogeneous doping using extrinsic donors [10,11,12,13,14], by modulating the charge carrier density via transistor-like configurations [15], and by generating two-dimensional electron gases, e.g., in LaAlO_3_/SrTiO_3_ structures or on modified STO surfaces [16,17,18,19,20,21,22], as well as by exploiting interface effects, e.g., in FeSe/STO structures [23,24,25,26,27]. In the literature dedicated to the transformation of stoichiometric STO (a band insulator) into a material with delocalized electrons [9], it is assumed that the doping induced by thermal reduction is homogenous throughout the whole crystal. Notably, Jourdan et al. [28,29] has already discussed an essential contribution of the surface layer to the electrical transport phenomena in this context. On the other hand, interdisciplinary studies have clearly presented that in the surface region of thermally reduced STO (prepared under the same conditions as for the transformation into a superconductor), oxygen vacancies are preferentially accumulated along the cores of dislocations [30]. A similar effect has been observed in electro-degraded STO at moderate temperatures of 200–500 °C [31]. This process results in a localized filamentary metallicity related to a network of dislocations and has been used to explain the nature of resistive switching in STO single crystals. Moreover, it has been found that in the low resistance state (the so-called “on” state of resistively-switched STO), the filaments can be transformed into nonstoichiometric titanium suboxides (Ti_n_O_2n−1_), so-called Magnéli phases [31,32,33,34]. It should be noted that the terms “metal” or “metallic state” are used in the following according to the definition by P.A. Cox [9], who classified metal oxides, in which band theory seems to provide a good description for most of their properties, as “simple metals”. However, reduced transition metal oxides might not fulfill all criteria of the conventional definitions of a metal.

Verifying whether the character of the transition into the metallic state in thermally-reduced STO is homogenous or filamentary is, in our opinion, the key to understanding the nature of the superconductivity in this prototype material. Therefore, our paper is dedicated to solve this problem. Despite the fact that in the literature about superconductivity, homogenous models of the distribution of carriers dominate [35,36,37,38,39,40,41,42], whereas a filamentary picture (accepting a non-homogenous incorporation of d^1^ electrons in the crystal) has been established in numerous papers discussing resistive switching [43,44,45,46,47,48,49,50,51,52,53], we are able to find a common denominator between the two mentioned completely controversial approaches. In both models, it has been accepted that the origin of the electronic carriers generated during reduction reflects the generation of ionized oxygen vacancies upon the thermally-induced removal of oxygen from STO. Therefore, our paper begins with a discussion of the thermal reduction process of STO in a vacuum. Then, we present the unique role of the surface region for superconductivity (as supported by Jourdan et al.’s idea [28]). Two questions will thus be answered. First, what is the accurate amount of oxygen vacancies generated by such a treatment, and second, what is their distribution? Note that we will not discuss the incorporation of oxygen vacancies after reduction in contact with a getter (e.g., metallic Ti) in detail, which is connected with an enormous lowering of the partial pressure of oxygen and, in consequence, leads to a stoichiometry polarization resulting in the creation of new Ti-rich phases in the surface region [54].

The primary methods by which the amount of oxygen removed from the crystal during reduction can be determined are based on either measuring the weight change in the crystal (thermogravimetric analysis, TGA) or the oxygen outflow (quadrupole mass spectrometry, QMS). These techniques allow comparison of the maximum concentration of electronic charge carriers introduced during the reduction (2 × *N*_e_ ≈ *N*_VÖ_, for twofold ionized oxygen vacancies) with an estimate of the charge carrier concentration from Hall measurements (where the uniformity of the current flow is assumed without any detection at the nanoscale). Using the primarily mentioned gravimetric method, we can also reliably calculate the number of oxygen ions that are released from the crystal during the electrochemical formation of insulating STO. This process is also called electro-reduction, electro-formation, electro-degradation, or electro-deoxidation, and is the first preparation step of resistive switching. Since electro-reduction can be realized at moderate temperatures (*T* < 450 °C), we can use this method to induce a filamentary insulator-metal transition and prevent the introduction of the oxygen vacancies in the matrix. Below 500 °C, an incorporation of oxygen vacancies in the crystal matrix is not to be expected from point defect chemistry. Hence, the transition process is only limited to the electroformed filaments. This allows us to analyze the similarities and differences between metallicity and superconductivity in STO after thermal and electrochemical reduction. Based on the detection of filamentary conductivity, we hope to clarify the 60-year-old mystery about the nature of the transition of a dielectric STO crystal into the superconducting state, which occurs at an extremely small amount of oxygen vacancies, and propose new conceptions (models) for a non-homogeneous transition.

## 2. Materials and Methods

### 2.1. Sample Preparation

Strontium titanate crystals (100) were purchased from CrysTec (Berlin, Germany). Samples with typical dimensions of 10 × 3 mm^2^ and thicknesses of 0.5–1 mm were prepared by means of wire saw cutting. The samples were then cleaned in an ultrasonic bath in acetone, deionized water, and ethanol. The electrical connections were established using thin Pt wires in a four-probe configuration [55] and, additionally, the contact sample and wires were pasted with a conducting Pt suspension. Pre-heating in a vacuum at 200–300 °C for 0.5 h was employed for removal of the organic solution in the Pt paste and the water occluded on the surface of the sample.

### 2.2. Thermogravimetric Measurements

In situ measurements were achieved with a thermo-balance (TG439, Netzsch, Selb, Germany) with a resolution of 0.1 µg. The reduction of the STO crystals was obtained under vacuum conditions for an oxygen partial pressure below 10^−10^ bar at constant temperatures (600–1100 °C, with steps of 100 °C). The weight change was related to a reference sapphire crystal. The typical sample weight was 100 mg. Before the measurement was started, the long-time drift of the thermo-balance was controlled using a further sapphire crystal as calibration in the full temperature range. The weight analysis for a sample with a frozen oxygen defect concentration after thermal reduction was conducted employing a sensitive balance (SE-2 Micro Balance, Sartorius, Göttingen, Germany). This measurement was carried out ex situ.

### 2.3. Thermal Reduction and Effusion Measurements

Thermal reduction was performed using a quartz tube furnace under ultra-high vacuum (UHV) conditions, and the effused oxygen was measured with a residual gas analyzer (e-Vision 2, MKS Instruments, Andover, MA, USA). The base pressure was 5 × 10^−10^ mbar. At first, the sample was pretreated in the cold zone of the tube at a temperature of approximately 300 °C in order to desorb water. Then, the oxygen effusion was recorded while moving the crystal mounted on a holder made from Pt wires from the cold zone into the hot region at a constant annealing temperature (e.g., 1000 °C) using a magnetic transfer system. The experiment was conducted across three steps. First, a background measurement was performed by measuring the partial pressures using QMS while moving the empty Pt sample holder from the cold into the hot zone. In the next step, the STO sample was measured at the same pressure and temperature as the reference measurement. In a third control step, the reduced sample was repeatedly moved from the hot zone to the cold zone and the oxygen partial pressure was measured. We observed only a background signal in the third step, which indicated that the deoxidation process in the second step had been completed. The difference in the oxygen spectra for this three-step measurement gave a realistic amount of effused oxygen. In order to test if the speed of the turbomolecular pump of the UHV apparatus remained constant during effusion, Ne and O gas were dosed in the chamber via calibration leaks. The measured Ne partial pressure during this test remained constant in an oxygen partial pressure range of 10^−8^–10^−5^ mbar, showing that the pumping speed did not change. The calibration of the relation between measured partial pressure as a function of time and the amount of effused oxygen was obtained by connecting a container filled with pure oxygen of a defined volume and temperature to the UHV system. This oxygen was released to the measurement chamber via a needle valve in order to hold the partial pressure of oxygen during pumping constant. The calibration was performed twice for two different oxygen partial pressures. In this way, the average sensitivity factor of our apparatus for oxygen effusion was determined. The maximal error of our measurements was 15–20%. Further details of the measurement technique can be found in [30,56].

### 2.4. Micro-Valdes Measurements

Four-probe resistance measurements were performed in so-called Valdes geometry [57] with a custom-made measuring head, in which the distance between the Pt/Ir electrode tips was 0.5 mm. The distance between the electrodes was controlled by optical microscopy. An alternating current (AC) system (aixDCA, aixACCT Systems, Aachen, Germany) with two electrometric followers was utilized for the electrical characterization. The typical AC amplitude was a few mV at a frequency of 172.5 Hz. The details of the AC system were presented in our previous paper [55].

### 2.5. Local Conductivity Atomic Force Microscopy (LC-AFM)

The LC-AFM measurements were performed under vacuum conditions at 10^−5^ mbar using a JSPM 4210 setup (JEOL, Tokyo, Japan). Conducting Pt/Ir-coated tips (PPP-ContPt, Nanosensors, Neuchâtel, Switzerland) were used and biased with a few millivolts. The LC-AFM maps were analyzed using WinSPM software, version 407 (JEOL, Tokyo, Japan). In order to reduce the noise level of the atomically-resolved conductivity maps, only a software low-pass filter was used (without FFT transformation), noting that on all of the LC-AFM maps with atomic resolution, the distribution of atoms was visible to the naked eye. In order to ensure that true atomic resolution was achieved, the LC-AFM data were only examined when the position of atoms (in the same region) was the same for several scans.

### 2.6. X-Ray Photoelectron Spectroscopy (XPS)

A XPS spectrometer using monochromatized Al-K_α_ rays (5600, Physical Electronics, Chanhassen, MN, USA) was employed with a specially constructed heating stage, allowing it to reach a maximal temperature of 1100–1200 °C in situ. An area with a diameter close to 1 mm was excited by the X-ray beam and the electrons were detected at a sample-detector angle of 45° with an energy resolution of 0.05 eV. The pressure in the main chamber at higher temperatures was in the range of 10^−9^ mbar. The furnace and heating elements did not include any getter material (W, Ta, Mo, Ti, Si, C). Temperature measurements were obtained with a type S micro-theromocouple and pyrometrically. 

### 2.7. Electro-Reduction Apparatus

Details of the aixDCA apparatus (aixACCT Systems, Aachen, Germany), for electro-reduction and resistance measurements, are provided in Rodenbücher et al. [55]. The pressure in the vacuum chamber during the reduction process was *p* < 10^−7^ mbar (the partial pressure of the oxygen was 2–3 orders of magnitude smaller than the base pressure, i.e., *p*_O2_ < 10^−9^ mbar). The sample was annealed at 350 °C and a voltage of 200 V, and a current compliance of 10 mA was applied. During the electro-reduction process, the potential drop between the inner electrodes was monitored electrometrically. In this manner, the total resistance of the sample (S), as well as the resistances of the bulk (B), anode region (A), and cathode region (C) were measured. For comparison, we also performed electro-reduction measurements in stepwise constant current mode with different currents of up to 10 mA leading to a higher initial voltage. For a polarization voltage higher than 200 V, the high ohmic input (Hi) of the electrometer (Keithley 6514, Keithley, Solon, OH, USA) was connected to a voltage divider (1:100) consisting of high-impedance resistors (10^11^ Ω/10^9^ Ω +/− 1%). In all of the electro-reduction experiments, the current was measured with a Keithley 6430 subfemtoampere source (Keithley, Solon, OH, USA).

### 2.8. Superconducting Properties Measurements

Electrical resistance measurements were performed for rectangular samples with dimensions of 1.0 × 3.1 × 10 mm^3^ using the physical properties measurement system (PPMS Model 7100, Quantum Design, San Diego, CA, USA) equipped with a 14 T superconducting magnet. The four-point technique was used to measure the long-term resistance, R, with a suitably high level of accuracy. Current leads (silver wires with a diameter of 0.1 mm) were glued with silver epoxy to the end faces of the samples in order to obtain a homogenous current distribution in the central area where voltage leads (silver wires with a diameter of 0.05 mm) were glued at a distance of l ≈ 5 mm, which varied slightly for individual samples. The obtained contacts had a resistance below 1 Ω for current leads and 20–50 Ω for voltage leads (~0.5 mm-wide silver-epoxy strips) and were stable over time. For the resistance measurements, the electric transport option (ETO) was utilized with an AC current of 18–21 Hz frequency and amplitude of 0.05–0.2 mA. For these conditions, the resistance in the normal state was frequency- and amplitude-independent. The resistance in the magnetic field was measured with increasing temperature for a fixed field (magnet persistent mode) or with increasing and decreasing fields for a fixed temperature. The temperature was changed at a rate of 0.02 K/min, for which no significant *R*(*T*) hysteresis was detected for the up and down sweeps.

### 2.9. Density Functional Theory (DFT) Simulation

We performed density functional theory calculations in the generalized gradient approximation [58], employing the full potential linearized augmented plane wave method [59]. In order to correct the band gap of SrTiO_3_, we used a Hubbard U correction, as proposed in [60]. For the simulation of the extended defect, three unit cells of SrTiO_3_ were removed from a 6 × 5 × 1 supercell with an additional oxygen vacancy in the lower left of the defect. The defect-induced states were energetically located at the bottom of the conduction band to estimate their spatial distribution, and the charge density was integrated into the atomic sphere of 1.9 Å around the Ti sites. The local polarizations in each unit cell were calculated from formal charges of +2 and −2 for Sr and O, respectively, and their relaxed positions in the Ti-centered unit cell. 

## 3. Results

### 3.1. Thermal Reduction of Stoichiometric STO

**Figure 1:**
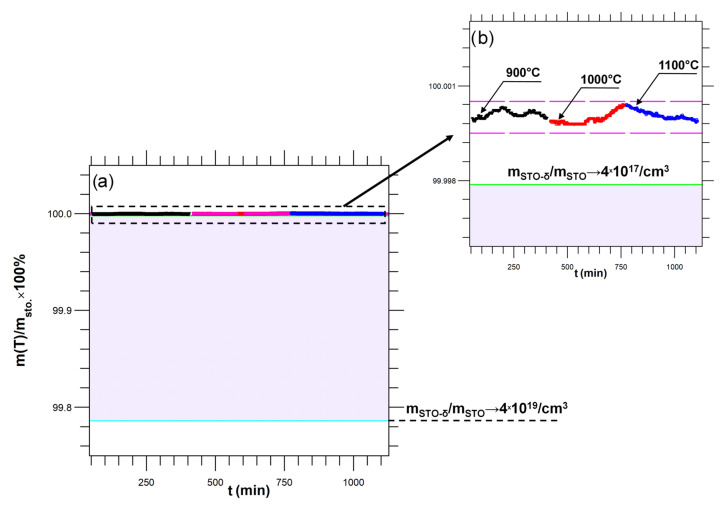
Mass loss of an STO crystal during thermal reduction performed subsequently at 900, 1000, and 1100 °C determined by thermogravimetric analysis ((**a**) overview, (**b**) magnification). The hatched area shows the hypothetical mass loss expected from the carrier concentration, measured by the Hall effect (4 × 10^17^–10^19^ carriers/cm^3^ [11]). The measured mass loss is below the resolution limit of the apparatus (±0.0005%), illustrating the discrepancy between the measurement and previous assumptions.

The thermogravimetric measurements of the oxygen non-stoichiometry induced by thermal reduction or electro-degradation of SrTiO_3_ → SrTiO_3−δ_ + ½ δO_2_↑ at first glance seem to be a suitable method for the determination of the amount of oxygen vacancies (δV_O_ = ½ δO_2_). A micro-balance is a typical tool for studying the effectivity of oxygen isotope exchange in STO. However, one needs 6 months to foster a complete oxygen exchange in a 0.3 mm thin crystal at 1000 °C [61]. In contrast, TGA studies of short-term reduction (0.5–1 h) of STO under vacuum conditions show that the mass changes at 1200 °C are extremely low, namely, 10^−4^ of the reference weight [62]. Additionally, when performing the reduction by using a flow of reduced gas (8% H_2_ in N_2_) at 1350 °C, a mass change of only Δm/m = 1.5 × 10^−4^ has been reported [63]. Although our TGA analysis allowed the measurement of a relative mass change in the order of 10^−6^, surprisingly, we were not able to identify any mass loss during the reduction of an STO crystal at 1000 °C under vacuum conditions (Figure 1). It has to be noted that with our resolution of mass change, we should have been able to detect mass losses related to carrier concentrations of 4 × 10^17^–4 × 10^19^/cm^3^, which have been reported by Collignon et al. [11] and references cited therein based on Hall measurements. The concentration of twofold ionized vacancies was two times smaller than the amount of electronic carriers. Therefore, the expected change of the weight between stoichiometric STO and reduced SrTiO_3−δ_ is Δm /m_stoich._ = 4 × 10^−5^ for a vacancy concentration of 2 × 10^17^/cm^3^ (green line in Figure 1), and Δm/m_stoich._ = 4 × 10^−3^ for a higher vacancy concentration of (2 × 10^19^/cm^3^). The measurements clearly demonstrate that despite the same reduction conditions as in references [1,8], our balance “did not budge”, although the (specific) resistance of our reduced crystal was smaller than the one referenced [1].

We attempted an additional ex situ experiment to verify the possibility of gravimetric identifications of the mass changes induced by thermal reduction at high temperatures and UHV conditions. After reducing the sample, the concentration of oxygen vacancies was frozen by quenching the crystal at an extremely fast cooling rate of 20–30 °C/s, a typical method for the constitution of metallicity or superconductivity in thermally-reduced STO. Using a micro-balance with a resolution of 0.1 µg, however, we did not detect any weight differences before and after reduction of an STO crystal at 800, 900, and 1000 °C. 

The analysis of the TGA data shows that this method is sensitive enough to falsify the proposed amount of oxygen vacancies based on Hall measurements, but not sensitive enough to determine the real concentration of oxygen defects introduced in the crystal upon reduction. Therefore, a spectrometric measurement with a quadrupole mass spectrometer is the only reasonable alternative for measuring the concentration of atoms released during the reduction processes. 

**Figure 2:**
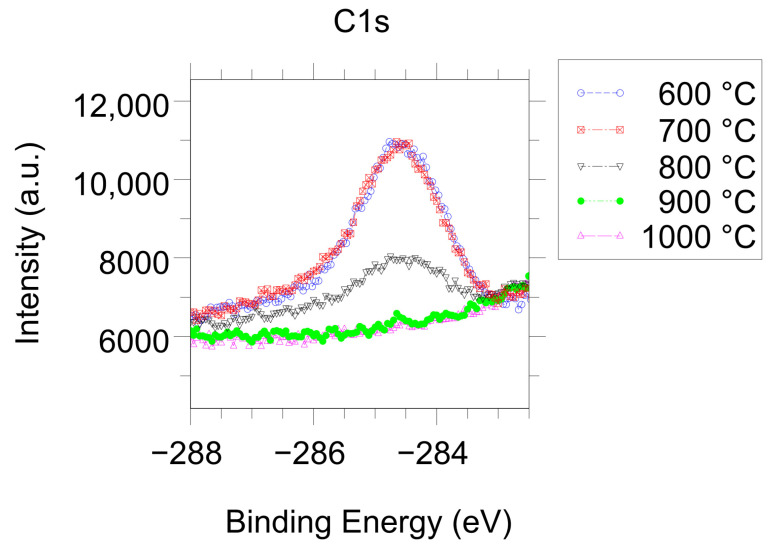
Operando XPS analysis of the C1s core line during reduction of an STO crystal under UHV conditions showing carbon removal from the surface above 700–800 °C.

The QMS measurement of the effusion processes during the reduction and electro-degradation of STO crystal has been obtained under UHV conditions with very low partial pressures of oxygen and OH/H_2_O in the measuring chamber. The apparatus was calibrated with a precisely determined amount of oxygen molecules. Therefore, a container with an exactly defined volume, temperature, and oxygen pressure was connected to the measurement chamber, and it was confirmed that the pressure when releasing the oxygen into the measurement chamber was in the measuring range of the QMS and did not change the pumping speed of the turbo-molecular pump. Additionally, information about the desorption of physi- and chemi-sorbates from the surface in UHV is necessary. For example, the product of calcination of SrCO_3_ (which is connected with the detachment of CO_2_ molecules from SrO terraces) at high temperatures and in vacuum conditions leads to the dissociation of CO_2_ → CO + ½O_2_ and the generation of atomic/molecular oxygen, which is not connected with the lowering of oxygen stoichiometry in reduced STO. Therefore, an operando study using X-ray photoelectron spectroscopy (XPS) was conducted for the same reduction temperatures. The analysis of the vanishing of the additional compounds of the O1s core line and the disappearance of the carbon oxides (see C1s line in Figure 2) admits an exact definition of the minimal temperature for which the surface of STO can be classified as clean according to surface physics rules. Thermal reduction above this temperature limit allows a clear correlation between the amount of outflowing oxygen (measured with QMS) and the formation of oxygen vacancies that have reduced the mass of the crystal.

Table 1 lists the oxygen removed during the thermal treatment at different temperatures. It should be noted that the values of 600 °C and 700 °C should be considered with caution, as they do not represent a pure concentration of oxygen vacancies in the crystal but are mixed with the desorbed oxygen from chemisorbates (here disassociated CO_2_). Despite the extremely small number of oxygen atoms generated by thermal reduction or electro-degradation, the crystal was transformed into a metallic state (see Table 1 and references [30,31,64]). Our QMS data, on one hand, show the impossibility of the detection of the mass loss using a balance and, on the other hand, illustrate the giant dissonance between the measured real concentrations of effused oxygen (which corresponds to the number of oxygen vacancies) and the carrier concentration determined using the Hall effect. This finding seriously calls into question the applicability of the magneto-effect for measuring the vacancy and carrier concentration of self-doped STO. In comparison to the insulator-metal transition induced by doping STO with aliovalent cations, e.g., La or Nb, the measured critical concentration for reduced or electro-degraded STO is 5–7 orders of magnitude smaller, which is a challenge for understanding the nature of this process. We must be aware that in both doping types, the electron transferred back to Ti either from the dopant cations (e.g., La^3+^ or Nb^5+^) or from the ionized vacancies ($V_{O}^{\cdot \cdot }$) is responsible for the increased electrical conductivity.

**Figure 3:**
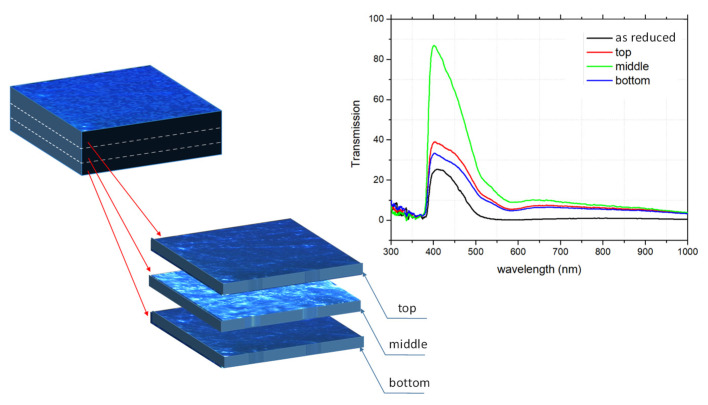
Investigation of the optical transmission of a reduced crystal. As schematically illustrated, the crystal was cut into three parts after thermal reduction (left). After cutting the crystal, all rough surfaces were epi-polished. The graph displays the spectral dependence of the normalized absorption for the whole reduced crystal (black), its middle part (green), and both parts including the surface regions (red and blue), illustrating that the dominant absorption comes from the surface regions.

So why do we need only such a low concentration of oxygen defects to “switch” the crystal to a metallic or superconducting state for both types of doping, despite the dominant role of d^1^ electrons? This paradox can be understood if we consider the crystal as an inhomogeneous object before and after the removal of oxygen. For the optimal thermally-reduced STO, the reduction time should be coordinated with the position of the resistance minimum of the reduction curve for each temperature (see [64]). Such a crystal achieves optimal electrical conductivity and changes its color to grey or dark blue. At this point, we need to accept that prolonged annealing under constant oxygen partial pressure does not lead to the establishment of an equilibrium state as could be expected from point defect chemistry [65]. Due to the self-healing effect, the prolongation of the annealing time renders the crystal more transparent and the electrical conductivity decreases again. This means we have only a short time interval for reaching a maximal reduction state of the STO crystal [18]. However, if we look closely at such a maximally reduced crystal, the transparency is not reduced in the whole crystal. As can be seen from the tomographic optical investigation presented in Figure 3, the major contribution to the absorption due to free d carriers introduced during the reduction process stems from the surface region. Only the plates containing the surface regions are responsible for the strong optical absorption. 

**Figure 4:**
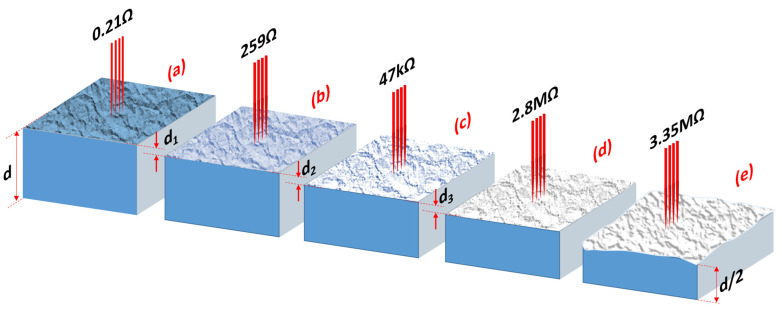
Illustration of resistance tomography measured with the micro-Valdes method for optimally reduced STO realized by subsequent mechanical removal of the surface (**a**–**e**). In each polishing step, a layer with a thickness of *d*_1_ = *d*_2_ = *d*_3_ ≈ 10 µm was removed. The major contribution to the global resistance comes from the last few dozens of micrometers-thin surface regions (see difference between (**a**,**b**)). The resistance of the reduced crystals determined after removal of 3 layers with a total thickness of about 30 µm (here 2.8 MΩ) is of the same order as after polishing half of the crystal (3.35 MΩ in (**e**), *d*/2 = 250 µm).

Another impressive proof of the dominance of the surface region of optimally thermally reduced STO in electrical transport can be provided by so-called resistance tomography analysis using the micro-Valdes method. Through the successive mechanical removal (polishing) of thin layers of approximately 10 µm thickness starting from the original surface of the reduced crystal, the contribution of polished areas to the total conductivity can be measured. Figure 4 shows that the upper part of the surface area (with a several µm thickness) contributes dominantly to the total conductivity, analogous to the optical measurements (Figure 3). This conclusion is in agreement with our in situ analysis of the surface of reduced STO obtained by the four-tip scanning tunneling microscopy (STM) method [66], which allows for calculation of the contribution of layers of different thicknesses in the surface regions to the total conductivity by modifying the distance between the tips. Furthermore, these measurements revealed that the character of the conductivity is not a homogeneous bulk conductivity, but is confined to the surface region. This behavior demonstrates the “spectator role” of the bulk in electrical transport. The optical absorption and resistance tomography measurements highlight the special role of the surface region. Still, they did not answer how this region can be switched into a metal or superconductor after the effusion of a very small amount of oxygen. Hence, we will take a closer look at this region in the following subsection.

### 3.2. The Dislocation Network in the Surface Layer of Epi-Polished STO

Due to the mechanical polishing of the surface of the STO crystal, a network of extended defects (especially edge dislocations) is introduced in a surface region with a thickness of approximately 30 µm. This network possesses a ranking character. The dislocation density is 10^10^/cm^2^ for epi-polished surfaces and 10^12^/cm^2^ for a rough surface (e.g., after cutting). In the deeper part of the surface region, the dislocation density reaches the original density of dislocations in the virgin crystal, 10^6^–10^7^/cm^2^ [31,67]. This progressive reduction in the density of dislocations with increasing depth automatically leads to the creation of a hierarchic tree; that is, the number of branches below the nodes is smaller than above the nodes (with respect to the surface of the crystal). Since the dislocations as extended defects underlie the crystallographic rules, the conservation of the Burgers vector, which describes the distortion of the lattice caused by a dislocation [68], is required for each node where dislocations meet, i.e., the sum of the Burgers vectors must be zero at this point. The existence of a network of dislocations in the perfect matrix of the dielectric SrTiO_3_ crystal complicates the description of the surface region not only from the crystallographic point of view, but also regarding changes in the local chemistry/composition, electronic structure, and polarization of the dislocation core (see investigations by transition electron microscopy (TEM), electron energy loss spectroscopy (EELS), and energy dispersive X-ray spectroscopy (EDX) [69,70,71,72,73]). As a result, the interconnected dislocations in the network cause an electrical short circuit of the insulating part of the crystal. 

**Figure 5:**
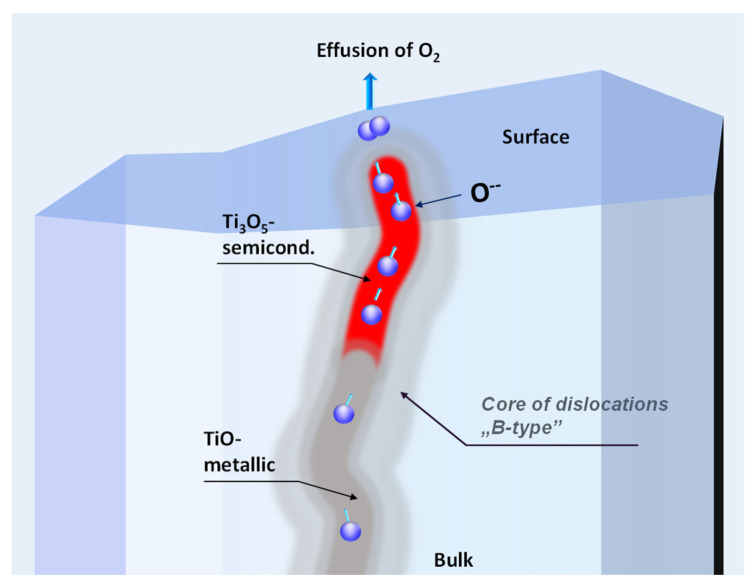
Illustration of a core of dislocation during the reduction process. Based on HRTEM, ChemiSTEM, and EELS investigations, the local chemical composition of a core has to be regarded as a combination of segments with different content of oxygen typical for low Magnéli phases or low Ti oxides (here TiO). At high temperatures and low partial pressure of oxygen, the maximum deoxidation rate occurs in the segment with highest oxygen stoichiometry (e.g., Ti_3_O_5_ [31]).

Now, with the information about the chemical composition of dislocations cores and their interrelated connection in the surface region, we can identify why an insulator-metal transition can be induced in the surface region at very low concentrations of oxygen vacancies, which are generated during thermal and electrochemical deoxidation of this native composite: bulk STO + a network of dislocations. The core of dislocations in stoichiometric STO possesses a composition similar to Ti_3_O_5_ [69,70,71,72,73], Ti_2_O_3_, or TiO, as provided by high-resolution TEM/EDX measurements. For such a chemistry of the core of dislocations, the local concentration of oxygen vacancies is approximately a few dozen per cent [70]. Because the segments along the dislocation’s line do not have continuously identical oxygen non-stoichiometry, the serial connections of metallic TiO are interrupted by, e.g., semiconducting Ti_3_O_5_. Therefore, in the original dielectric STO substrate, the set of dislocations as a whole do not produce a metallic short circuit, although fragments of dislocations with metallic properties exist (see the comparison between the macroscopic resistance of the stoichiometric STO and nanoscopic investigations using LC-AFM [31]). During the thermal or electrochemical deoxidation, the segments of higher TiO oxides will be preferentially reduced (see thermodynamic rules for reducing TiO oxides [31]). In this way, the semiconducting fragment of dislocations becomes metallic (Figure 5) and can create a continuous metallic short circuit with the other metallic pieces. This behavior is essential for understanding why our nano-composite in the surface region can be transformed into a metallic system and, consequently, a superconductor for this extremely low effusion of oxygen. Of course, it could be assumed that the position of the segments with metallic properties may be arbitrary (e.g., the TiO metallic segment could be close to the surface of the crystal, as depicted in Figure 5). In this case, the problem arises as to whether it would be possible to diffuse oxygen through the low Ti oxides region with delocalized d electrons. Marshall et al. have impressively demonstrated that, for low Ti oxides, where oxygen vacancies are an integral part of the structure, the hopping of this point defect can occur at higher temperatures [74] with similar enthalpy as for oxygen in STO. 

**Figure 6:**
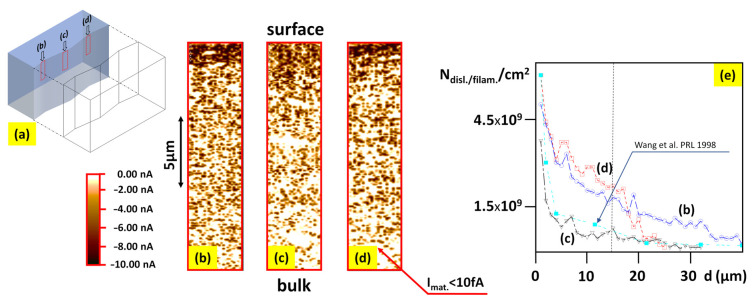
LC-AFM cross section analysis of a thermally reduced STO crystal. The mapping on a cleaved plane has been obtained in three positions as schematically depicted in (**a**). The out-of-plane distribution of filaments (conducting dislocations) in different positions (**b**–**d**) shows the hierarchic character of the dislocations tree/network in the upper part of the surface region. The change in filament density as a function of distance from the crystal’s surface indicates a similar trend as in the dislocation distribution in the surface region investigated with TEM (cyan curve in (**e**)) [67]. To record LC-AFM mapping of the deeper parts of the surface region, the position of the tip was shifted by scanning (using a micro-table) by about 17–18 µm relative to the previous mapping; in this case, for a scanning area of 20 × 20 µm^2^, it was possible to “seamlessly” connect two or three maps.

To not repeat experimental data about the thermal reduction of STO and the role of dislocations in this process, we only list the following properties with appropriate literature references:

1. Thermal reduction is limited to the core of dislocations (see the correlations between position etch pits and the position of the conducting filaments [30,31,64,75]). Significantly, during this reduction, the segment of dislocations with higher Ti oxides increases the oxygen non-stoichiometry more easily, i.e., it is more effectively reduced. The resistance of conducting dislocations in stoichiometric STO is lowered by six orders of magnitude after reduction, although the dislocations are segmented with metallic TiO or semiconducting Ti_2_O_3_ nanophases [31].
2. The area between conducting dislocations (the so-called matrix) of reduced STO possesses the same electrical resistance as in a stoichiometric crystal (resistance of 10^15^ Ω [30,31]).
3. The concentration of the conducting filaments and their distribution (LC-AFM mapping in-plane) is similar to the density of dislocations and their distribution determined by the TEM and etch pits technique [30,31].
4. For the cross-section of thermally reduced STO (Figure 6), the correlation between the distribution of conducting filaments (LC-AFM) and the change in dislocations density (TEM) has been given in [31].
5. The properties presented in points 3 and 4 allow us to analyze the dislocation distribution in the surface region with a thickness of 30–40 µm as a hierarchic tree of extended defects and, from an electrical point of view, as a hierarchic network of galvanically conducting dislocations [30].
6. From nanoscopic measurements of the integrated electrical conductivity of the edge dislocations, the macroscopic metallic conductivity can be derived [31]; the rest of the crystal plays only the role of “spectator” for the electrical transport, although it is a supporter of the three 3D metallic networks of dislocations.

**Figure 7:**
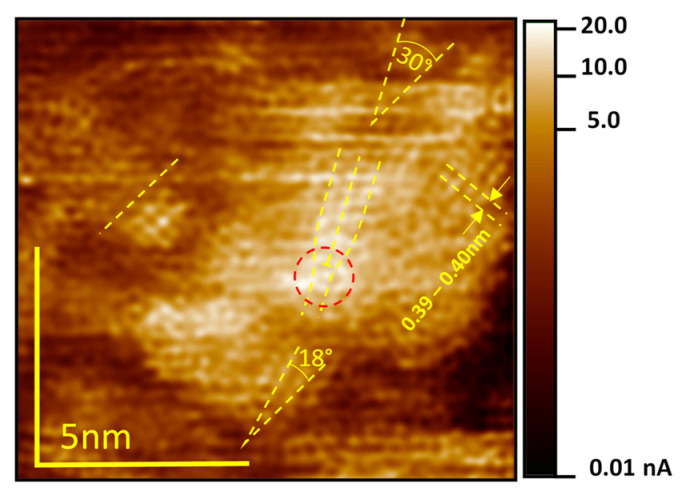
LC-AFM map of the electrical conductivity near a bundle of filaments. The red circle marks the conductivity increase close to the dislocation’s position. The change in the orientation of the blocs around the highly conducting area (see the different twisting of the orientation of the rows of atoms marked with yellow lines; here, 18° and 30°) suggests a kind of structural transformation into low TiO oxides. During scanning, a polarization of only 2 mV was used. The data are original data, and only a low-pass filter was used for the reduction of high frequency noise. A similar atomic resolution was observed for many scans and different regions of thermally reduced crystals.

In order to illustrate the highly inhomogeneous conductivity of thermally reduced STO, we performed LC-AFM as depicted in Figure 7. A bundle of conducting filaments, whose conductivity decreases rapidly at a distance of 1–2 nm, can be identified at the boundary (see lower right corner). This demonstrates that outside the agglomerated filaments, the conductivity is very low. Since the internal transimpedance amplifier of our LC-AFM having a sensitivity in the picoampere range does not allow analyzing the low conductivity between the bundles, we have used an external highly sensitive current-to-voltage converter for the determination of the resistance in this part of crystal, which is free of filaments. In this area, the conductivity is, in fact, similar to stoichiometric crystals, i.e., the local resistance under the AFM tip is in the range of 10^12^ Ω (see [31]). This discovery also confirms the role of the bulk as a “spectator” in electrical transport, even in reduced crystals. 

Finally, after this comprehensive analysis of the macroscopic and microscopic properties of the network of dislocations in the surface region, we are now in a position to calculate the average local concentration of the charge carriers introduced by the thermal reduction along the core of dislocations (Table 2). Therefore, the increase in oxygen non-stoichiometry in the network at each temperature step was calculated by assuming that the total effused oxygen (taken from Table 1) only originated from a network of dislocations in the surface region with a volume of 1 × 1 × 0.003 cm^3^. With the dislocation density estimated by Wang et al. [67], this resulted in a total length of dislocations of 2.6 × 10^6^ cm. For calculating the total volume of dislocations network, it was assumed that the HRTEM radius of dislocations core was equal to 10^−7^ cm.

The value of the local concentration of the carriers introduced by the selective generation of oxygen vacancies along extended defects in the hierarchic network of dislocations is similar to the doping level of La or Nb (of a few per mille) necessary “to switch” the stoichiometric STO into a metallic conductor and consequently into a superconductor [11,12]. In other words, our analysis suggests not only a giant heterogeneity in the electrical transport phenomena in reduced STO crystals (with a dominating contribution to the conductivity from preferentially reduced cores of dislocations), but also questions the existence of the part of the superconducting dome used for the creation of the models for dilute doping in SrTiO_3-δ_. 

### 3.3. Stability of the Surface Region upon Heavy Thermal Reduction

In transforming stoichiometric STO crystals via thermal reduction into a metallic or superconducting state, four factors play an essential role: the reduction temperature, the oxygen activity, the time of the exposition on reducing conditions, and the quality of the crystal [64]. 60 years after the discovery of semiconducting properties of thermally reduced STO [1], one would expect that this transformation would be completely understood and allow for tailoring the required electrical properties of the crystal by choosing appropriate reduction conditions. Nothing could be further away from the truth. It was reported that even when treating different samples from the same batch with identical geometry under identical reduction conditions, their resistance can vary by many orders of magnitude (e.g., see the paper by Spinelli et al. [8]). In the literature about thermal reduction of STO, one can find that such a treatment has been obtained at a broad range of temperatures (from 600–1400 °C) and different reduction times spanning from 1 to 20 h [8,76,77,78,79]. 

**Figure 8:**
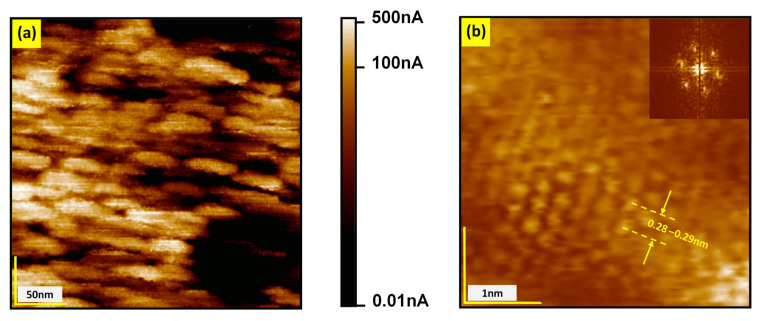
LC-AFM mapping (polarization voltage 10 mV) of a part of a strongly reduced STO crystal. (**a**) Current distribution on the nanoscale; (**b**) magnification of a highly conducting region with atomic resolution showing that the lattice constant and the symmetry (see fast Fourier transform in the inset) of the conducting island has been significantly transformed compared to stoichiometric STO.

Reduction under vacuum conditions, which can be generated by fore-vacuum pumps, turbomolecular pumps, or ion-getter pumps, allows for the introduction of oxygen vacancies in the system (in most cases, it can be found in the literature that the reached vacuum is in the order of 10^−6^ mbar). Unfortunately, the term “vacuum” does not contain information about the partial pressure of oxygen, a critical parameter for reaching the point defect equilibrium. The two other methods used to reduce oxygen activity are connected with reducing gases (H_2_ or gas mixtures of CO/CO_2_) or metallic getters. In this first case, information about the partial pressure of oxygen allows reduction under defined conditions, but with one small caveat: the temperature used in such a treatment is extremely high (from 1000–1400 °C) [62,63]. This high reduction temperature introduces oxygen vacancies and completely changes the surface layer’s chemical composition. Our in operando XPS studies showed that the surface layer transforms into Ti-rich phases (the ratio Sr/Ti is lowered from 1 to ~0.05 and the dominant Ti valence is 3+ and 2+ [31]). Under extremely reducing conditions (*p*_O2_ < 10^−17^ bar), which can be established by a Ti getter or electrochemical stoichiometry polarization and high temperatures of 1100–1250 °C, even the oxide Ti_3_O can be created. Note that for a Ti_2_O single layer, the transition into the superconducting state at 9.8 K has been reported already [80]. Our LC-AFM mapping of strongly reduced SrTiO_3_ presented in Figure 8 reveals that the electrical conductivity increases, and the symmetry changes to hexagonal, which is, for example, typical for Ti_2_O. Therefore, regarding superconductivity, such a heavily reduced region can be analyzed as TiO_x_ or Ti_y_O with a typical superconducting dome, in which *T*_C_ is a function of the oxygen content [81]. 

### 3.4. Electroreduction of STO

**Figure 9:**
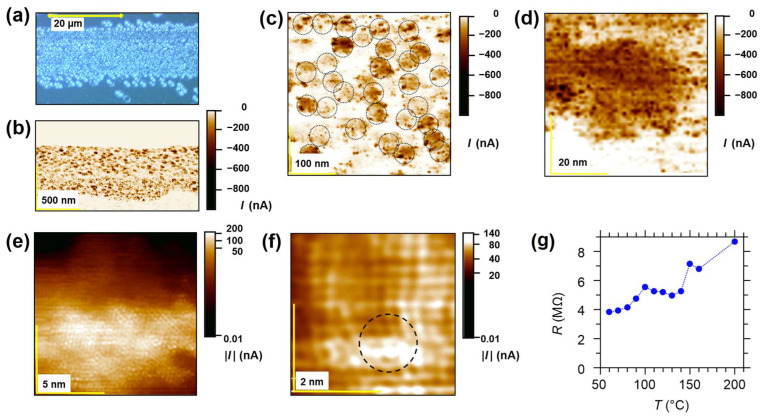
Detailed analysis of the dimension of the filaments. (**a**) Microscopic inspection of the etch pit distribution; (**b**) in-plane LC-AFM map obtained near the cathode (U_sample_ = −0.01 V); (**c**) magnification of (**b**) with filament bundles marked by a black dashed line; (**d**) magnification of a typical bundle; (**e**,**f**) LC-AFM maps of a nanofilament obtained with atomic resolution (the black dashed circle marks the region with highest lattice distortion and conductivitiy); and (**g**) temperature dependence of the resistance, measured by placing the LC-AFM tip above a nanofilament.

In order to enhance the filamentary network of conducting dislocations, electro-reduction was performed. This method allowed the induction of well-defined dislocation-based metallic filaments (for details, see Supplementary Information). During this process, a short circuit associated with orthogonal dark bands in [100] orientation was generated between the anode and cathode [82]. Within the dark bands connecting the anode with the cathode, an alignment of dislocations can be seen from the etch pits analysis (Figure 9a). Similar to the thermally reduced crystals, it was shown that the oxygen effusion during electro-reduction is extremely low, and only at the filaments is a carrier concentration high enough for metallic conductivity present [82]. Within the bands, an increased conductivity was observed by LC-AFM, confirming the correlation between dislocations and filamentary conductivity (Figure 9b). From the magnified LC-AFM maps (Figure 9c), it is apparent that the bands consist of bundles with a 40–50 nm radius, containing more than two hundred highly conducting filaments with a radius of approximately 2 nm each. 

Between the highly conducting nanofilaments, an increased conductivity can be observed (Figure 9d), which is one to two orders of magnitude lower than in the center of the filaments, but still significantly higher than in the insulating matrix. The distance to the neighboring nanofilaments was less than 3–4 nm for most of the filaments in the bundle. This suggests that the nanofilaments act as collective electron sources, doping the region between themselves. Close to the exit of a dislocation, whose position can be identified by analyzing the course of the atomic rows, the local conductivity reached a maximum (Figure 9f). In contrast, the conductivity between a nanofilament and the matrix at the edge of a bundle was three orders of magnitude smaller within only 1 nm (Figure 9e). In addition, the resistance of selected sets of bundles measured by LC-AFM (Figure 9g) increased with temperature, thus indicating metallic behavior. 

### 3.5. Simulation of the Electronic Structure

**Figure 10:**
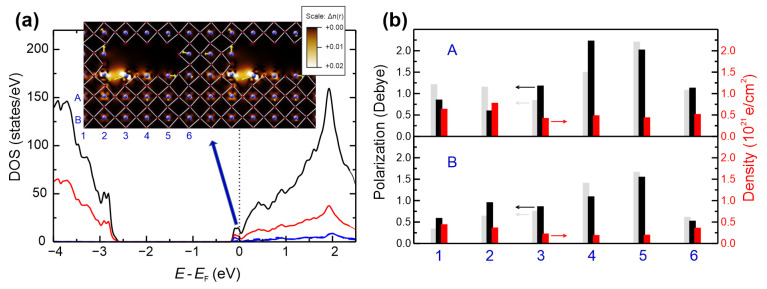
DFT simulation of neighboring one-dimensional extended defects in STO. (**a**) Illustration of the electronic structure. The inset shows the relaxed structure with oxygen atoms marked in red and Ti atoms in blue. The vacancy-induced states are visible in the density of states (DOS) at the bottom of the conduction band. The total DOS is shown in black, the local DOS on the Ti atoms adjacent to the vacancy in blue, and the interstitial DOS (between the atomic spheres) in red. A cut through the induced charge density is shown in the inset underlying the atomic structure. Bright areas indicate a high electronic density. Large local polarizations are indicated by yellow arrows. (**b**) Polarization (black) and defect-induced charge densities (red) in STO near the extended defect. The vertical and horizontal positions of the Ti atoms are labelled with (A, B) and (1, …, 6), respectively, and are visualized in the inset of (**a**). For comparison, the size of the local polarizations in an extended defect without the oxygen vacancy are indicated by the grey bars.

In order to shed some light on the nature of metallic filaments forming in an insulating perovskite matrix, we performed DFT calculations (for computational details see Section 2.9) of extended defects (Figure 10a). Although quite simplified, this model captured two important aspects: (i) a one-dimensional, stoichiometric defect creates an “inner surface” where vacancies can be easily formed, and (ii) an oxygen vacancy (*V*_O_) at this inner surface leads to the formation of a defect state at the bottom of the conduction band, i.e., Ti d-states are available around the Fermi level, electronically similar to lower Ti oxides [83]. As the formation energy of *V*_O_ is lower at an (inner) surface than in the bulk [84], the accumulation of such defects at inner surfaces is likely. In addition, tendencies for the clustering of these defects have been reported [85]. The charge density of the defect state is primarily located on the nearby Ti ion (the blue DOS curve in Figure 10a) and in the region between the atomic spheres (red line). In Figure 10b, we estimated the charge density locally by integrating the induced defect charges in the Ti atomic spheres of the individual unit cells (red bars). A charge density in the order of 5 × 10^20^ electrons per cm^3^ extends into the region between two such extended defects (columns 1 and 6). This defect charge density decays quickly in the matrix, but larger conductive areas can form in a bundle of one-dimensional defects. 

We investigated how these local charges influence the local, static polarizations, as the formation of polar regions in combination with a conductive region will be essential for superconductivity. To estimate this quantity, we employed a simple ionic model based on the formal charges and the positions of O (***r***_O_) and Sr (***r***_Sr_) relative to Ti ions, calculating $P_{\mathrm{Ti}}=\sum \left(r_{\mathrm{Sr}}/4-r_{O}\right)$. Yellow arrows indicate these polarizations in the inset of Figure 10, and their norm is summarized in Figure 10b (black bars). We compared them to the polarization of a stoichiometric extended defect without *V*_O_ (grey bars). As is apparent, a small electronic density (below 5 × 10^20^) does not quench the local polarizations (of course, some reconstruction also results from the presence of the additional defect), and larger densities (position A1 and A2) can lead to some local reduction of ***P***_Ti_, i.e., high densities are anticorrelated with the polarization.

### 3.6. Measurements of the Superconducting Properties

**Figure 11:**
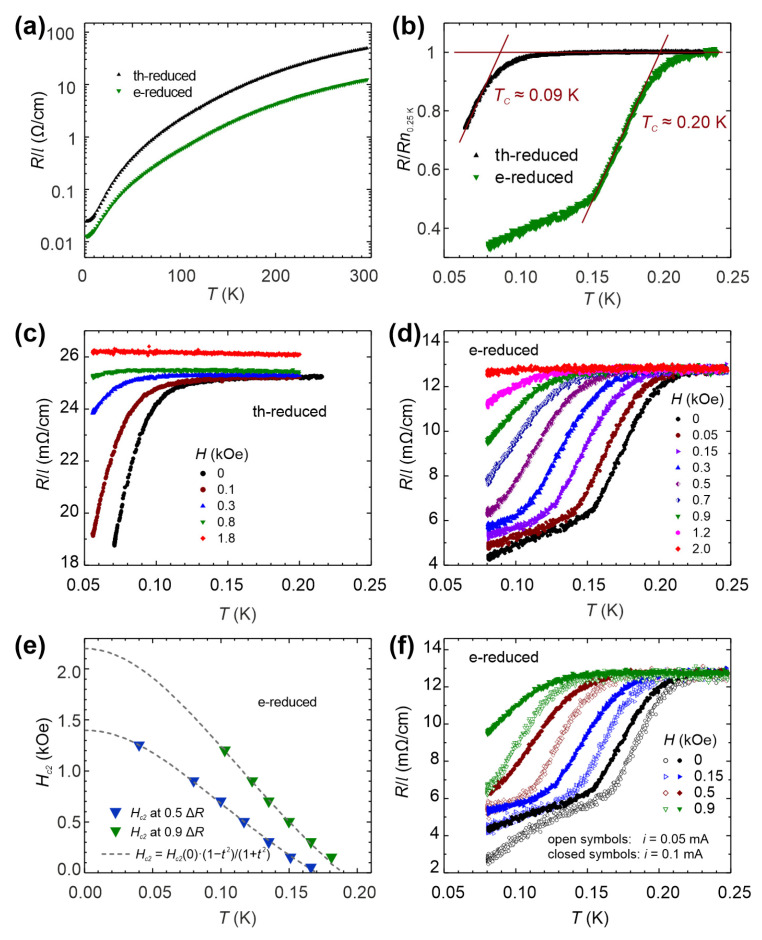
Resistance measurements of the thermally and electro-reduced STO samples. (**a**) Resistance, *R*/*l*, vs. temperature for the thermally (th-reduced) and electro-reduced (e-reduced) STO samples with the same cross-sectional area, where *l* is the distance between the voltage leads; (**b**) *R*/*Rn*_0.25K_ ratio for the thermally and electro-reduced STO samples below the superconducting transition temperature *T*_C_ (*Rn*_0.25K_ is the value of *R* in the normal state at 0.25 K); resistance, *R*/*l*, vs. temperature for (**c**) the thermally reduced and (**d**) the electro-reduced STO samples measured in different magnetic fields at a current of *I* = 0.1 mA; (**e**) upper critical field, *H_c_*_2_, vs. temperature for the electro-reduced STO sample. The results were obtained using the data displayed in (**d**) with the criterion 0.5Δ*R* (blue triangles) and 0.9Δ*R* (green triangles), where Δ*R* is the change in the resistance at the transition to the superconducting state. The dashed lines show the fitting of the experimental results to the equation *H_c_*_2_(*T*) = *H_c_*_2_(0)(1 − *t*^2^)/(1 + *t*^2^) where *t* = *T/T_c_*; (**f**) resistance, *R*/*l*, vs. temperature for the electro-reduced STO sample measured in different magnetic fields and at two measuring currents, *I* = 0.05 mA (open symbols) and *I* = 0.1 mA (closed symbols), for comparison.

We analyzed the superconducting properties of electro-reduced STO via low-temperature conductivity measurements in a magnetic field. For comparison, we also investigated a thermally-reduced crystal, in which a network of nanofilaments was not as pronounced and bundled as for electro-reduced STO. We utilized samples from the same crystal to ensure comparability (see Supplementary Information). For the thermally and electro-reduced STO samples, the temperature dependencies of the resistance were measured (Figure 11a). The resistance was divided by the distance between voltage contacts, *R*/*l*, such that the linear resistance characterizing a given material was obtained for samples with the same cross-section. As is evident from Figure 11a, *R*/*l* decreased with temperature, designating metallic properties. The resistance of the electro-reduced sample (with larger density of metallic nanofilaments) was significantly lower than that of the thermally reduced one, confirming that the metallic properties of deoxidized STO are, as we demonstrated, confined to the nanofilaments. At temperatures below 0.25 K, both samples exhibited a sudden drop in the resistance, which is characteristic of superconductivity. In Figure 11b, the resistance *R* normalized to the normal-state resistance at 0.25 K, *Rn*_0.25K_, is presented near the transition temperature, *T*_c_. The superconducting transition temperature for the electro-reduced sample (*T*_C_ ≈ 0.2 K) was more than a factor of two larger than that for the thermally-reduced sample (*T*_C_ ≈ 0.09 K), suggesting that superconductivity in the electro-reduced sample is caused by the bundled nanofilaments, and therefore enhanced by their increased density. An increase in *T*_C_ with the volume of the superconducting phase has been observed for “nano-sized” materials, e.g., in In and Pb nanoparticles [86] and Mo-Ge nanowires [87], with sizes comparable to the diameters of the nanofilaments (2–5 nm) and filamentary bundles (40–50 nm) found in our samples. Note that in the “superconducting” state, none of the samples exhibited zero resistance, which serves as another strong indication for non-percolative filamentary superconductivity (spatially-separated phase-coherent superconducting regions) [88] in deoxidized STO.

The behavior of *R*/*l*(*T*) in a magnetic field for the thermally and electro-reduced STO samples is shown in Figure 11c and Figure 11d, respectively. The results prove the superconducting nature of the transition to a lower resistance state and allow the temperature dependences of the upper critical field, *H_c_*_2_(*T*), to be determined. The criteria 0.5Δ*R* and 0.9Δ*R* were used, where Δ*R* is the change in resistance upon entering the superconducting state. Figure 11e shows the *H_c_*_2_(*T*) results for the electro-reduced sample, with the fitting of the experimental data to equation *H_c_*_2_(*T*) = *H_c_*_2_(0)(1 − *t*^2^)/(1 + *t*^2^), where *H_c_*_2_(0) is the value of the critical field at *T* = 0, *t* = *T*/*T*_C_, and *T*_C_ is the superconducting transition temperature at *H* = 0. Depending on the criterion, 0.5Δ*R* or 0.9Δ*R*, the coherence lengths *ξ*_0_ ≡ *ξ*(*T* = 0) = [*ϕ*_0_/2π*H_c_*_2_(0)]^0.5^ ≈ 49 nm and 39 nm were obtained for the values *H_c_*_2_(0) ≈ 1.4 kOe and 2.2 kOe, respectively, extracted from the fitted *H_c_*_2_(*T*) dependences. The linear extrapolation of *H_c_*_2_(*T*) to *H_c_*_2_(0) from the last experimental point in the high fields gives *ξ*_0_ ≈ 47 nm and 35 nm, depending on the criterion, which are lower limits for *ξ*_0_. 

In order to test which of the criteria (0.5Δ*R* or 0.9Δ*R*) provides more correct *H_c_*_2_(*T*) values, *R*(*T*) for the electro-reduced sample was measured at two different currents, 0.05 mA and 0.1 mA, and the results are shown in Figure 11f. For both currents, the transition to the superconducting state began at the same temperature, but for *I* = 0.1 mA, the transition was significantly broader, indicating an influence from the vortex dynamics. In order to minimize this effect, the more appropriate 0.9Δ*R* criterion was used to extract *H_c2_* (0) ≈ 2.2 kOe, *ξ*_0_ ≈ 39 nm, and *T*_C_ ≈ 0.20 K. A coherence length of *ξ*_0_ ≈ 40–50 nm is typical for conventional low-temperature bulk superconductors, e.g., Hg, Nb, or In. Moreover, the value of *ξ*_0_ ≈ 40 nm approximately corresponds to the diameter of the filament bundles, 40–50 nm, as obtained from the LC-AFM studies (see Figure 9), supporting our assertion that superconductivity, as well as the metallicity of STO, is filamentary in nature and appears in the reduced cores of dislocations.

### 3.7. Theoretical Description of Superconductivity

Generally, electron/hole pairing depends on the strength of the electron–phonon interaction λ, which is the product of the density of states and the electron–phonon interaction potential. This quantity must be larger than the Coulomb repulsion in order to produce pairing. In our low carrier density system, the Coulomb interaction is almost negligible within the insulating matrix, but might have some relevance, even though minor, in the metallic filaments [1]. For the insulating matrix, it is obvious that the density of states is too low to cause any feasible pairing, whereas in the filaments it achieves values comparable to other oxide superconductors; however, this interaction is not strong enough to cause superconductivity. This implies that the electron lattice interaction must be unconventionally strong, which immediately suggests polaron formation. It needs to be emphasized here that a very strong polaronic coupling leads to localization relevant to the matrix and causes polar properties, whereas a too small coupling causes band formation and delocalization. The intermediate coupling regime is the interesting one, which can mediate superconductivity. Interestingly, in our case, we are dealing with two components [10], namely, extremely strong coupling in coexistence with intermediate coupling. In addition, domains and domain walls play a crucial role, but are not considered in the following [89]. The interplay between strong and weak coupling regions has been shown to be the cause of electron pairing even if one channel (namely the insulating one) is, on its own, not superconducting, but is dragged into a superconducting state by the metallic one due to interband interactions. Theoretically, the polarizability model [90,91] and DFT calculations have been employed to confirm the heterogeneous character of doped STO. The polarizability model is based on the nonlinear polarizability of the oxygen ion, i.e., O^2−^, which in a crystal is stabilized by the surrounding ions. This implies that only the Ti ions, which adopt the d^1^ configuration, are affected in doped or electro-degraded STO. In turn, the surrounding O^2−^ ions are repulsed and cause locally strongly distorted regions. The essential task for both approaches is, however, to demonstrate the coexistence of a polar matrix with regions of different chemical composition. By concentrating on the dynamic properties of STO and knowing their development with carrier concentration *n*, the STO-related double-well potential has been shown to change its character from double- to single-well with *n*, where *n*_c_ defines the border line for this change [75,92]. As for *n* > *n*_c_, the superconductivity vanishes and a global metallic state is realized, and we only concentrate on the region *n* < *n*_c_.

**Figure 12:**
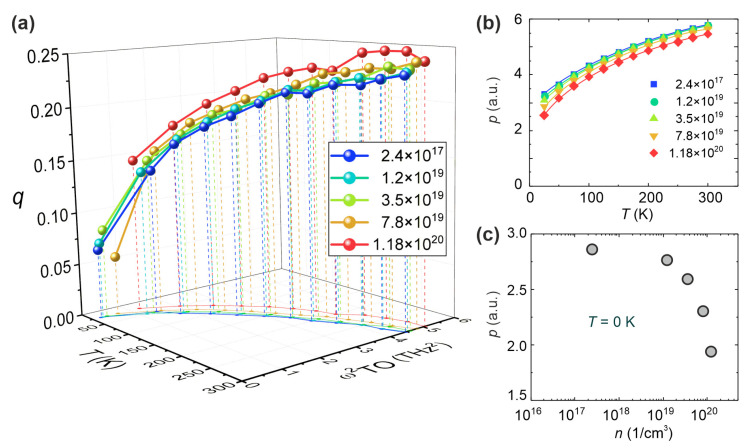
Lattice dynamics calculation of STO. (**a**) Temperature and momentum *q* dependence of the squared soft optic mode frequency $\omega_{TO}^{2}$ of STO for different carrier concentrations, as indicated by the color code in the figure; (**b**) temperature dependence of the local dipole moment p for different carrier concentrations; and (**c**) dependence of the dipole moment *p* on the carrier concentration *n* for *T* = 0 K [97].

The essential phonons in STO are the lowest lying soft transverse optic and acoustic modes [4]. Interrelation between these two branches is the signature of the formation of local polar nano-regions. By calculating the phonon group velocities for the two considered branches [93], these become very apparent and yield the momentum at which the local, spatially-confined soft modes occur, and where the scattering between the two modes is the strongest [94]. The corresponding squared polar optic mode frequency $\omega_{TO}^{2}(q)$ is displayed in Figure 12a as a function of carrier concentration *n*, temperature *T*, and momentum *q*. As is obvious from the figure, this local polar mode softens with decreasing temperature and moves to lower momentum values, but never reaches the homogeneous *q* = 0 limit [75]. In addition, the softening is reduced with increasing carrier concentration [95], and its momentum space decreases, indicating the growing spatial confinement and the shrinking of the polar nano-domains. In contrast to a long wavelength “true” soft mode, it is nonlinearly dependent on temperature below ≈150 K. As $\omega_{TO}^{2}(q)\propto \frac{1}{\varepsilon_{0}}$, the dielectric permittivity *ε*_0_ is extracted from it, and is approximately 40% smaller than the long wavelength limit, but still temperature dependent, typical for an almost ferroelectric compound [96].

From the calculated thermal average of the displacement-displacement correlation function, a local dipole moment has been derived that shrinks with decreasing temperature and increasing carrier concentration (Figure 12b). The zero temperature limit as a function of the carrier concentration is shown in Figure 12c. As expected, it rapidly decreases with increasing carrier concentration and approaches a constant value for small densities, supporting the polar character of the matrix. The carrier concentration range is compatible with our estimates from the DFT calculations shown above. In the other extreme, when *n* gets too small (<10^16^/cm^3^, see above), Cooper pairs cannot form any more. Thus, superconductivity can be observed only in a concentration range around 10^17^–10^19^/cm^3^.

The above analysis demonstrates that in the metallic and superconducting low carrier density limit of STO, metallicity appears in filaments that coexist with elastically and polar-distorted domains, where the latter shrink in size with increasing carrier density. These have vanished beyond a critical carrier density and homogeneous non-superconducting metal forms. Thus, the electronic band structure consists of localized insulating polaronic bands attributed to the matrix, whereas Fermi liquid-type behavior must be present in the filaments. With increasing carrier density, the localized band adopts dispersion from the itinerant one, analogous to a steep band/flat band scenario [98] in which superconductivity is a consequence of interband interactions.

## 4. Discussion

Our interdisciplinary analysis has shown that thermal reduction, which is frequently used to generate metallicity or superconductivity in STO crystals, is limited to a network of dislocations in an approximately 30 µm-thick surface region. Due to the preferential removal of oxygen from the core of dislocations, their fragments with higher Ti oxides are switched into metallic nano-conductors. The global effusion of oxygen during this process is extraordinarily small (only 10^13^ effused oxygen atoms from a standard STO substrate with a size of 1 × 1 × 0.05 cm^3^). In this way, the hierarchic network of dislocations adopts metallic and superconducting properties. In the analysis of the nature of the transition into the mentioned states, it is important to separate the moderate reduction, which allows us to consider the system as STO + nano-filaments, from strong reduction (above 1000 °C) or from thermal treatment in the presence of a getter, since these procedures change the chemistry of the surface layer completely. For both types of reduction, one cannot consider the crystal as a homogeneous object and determine parameters such as specific resistance/conductance or the concentration of the carriers per volume as is frequently performed in the literature, as one has to consider the dislocation-based filamentary nature of the conductivity.

**Figure 13:**
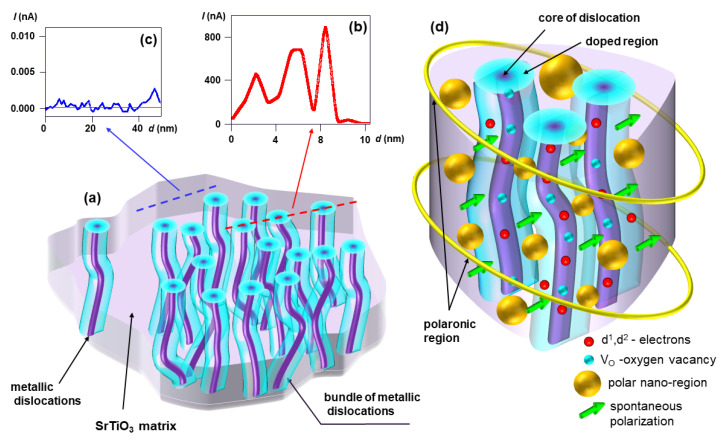
Schematic picture of the dislocation-based filamentary superconductivity in electro-reduced STO. (**a**) Arrangement of bundled metallic dislocations according to LC-AFM and etch-pit studies; (**b**) LC-AFM line profile across the dislocation cores, revealing that they have an extremely high conductivity; (**c**) LC-AFM line profile in the region without electro-reduced dislocations revealing insulating behavior; (**d**) illustration of the proposed coupling mechanism of pairs of d^1^ (and, possibly, d^2^) electrons in metallic dislocations mediated by a polar nano-regions and the spontaneous polarization (here, flexo or ferroelectric polarization) between.

In our paper, we have presented a broad spectrum of arguments supporting the idea that the dislocations in a moderately thermally reduced STO crystal are the origin of metallicity and superconductivity. To emphasize the relation between superconductivity and the evolution of filaments, we have compared thermally-reduced crystals with electro-reduced ones. Our results have shown that the electro-reduction of STO at moderate temperatures (*T* < 400 °C) induces superconductivity with a *T*_C_ ≈ 0.2 K, which is significantly higher than that of thermally-reduced STO (*T*_C_ ≈ 0.09 K). During electro-reduction, a dislocation-based network of filaments is generated (Figure 13a), which is more bundled (see Figure 9c) than for the thermally reduced case (see Figure 6b–d) and can more effectively channel the current flow via formation of a metallic short circuit. Our LC-AFM investigations proved that the conductivity of the filaments is orders of magnitude higher than that of the surrounding matrix (Figure 13b,c). We present a nanoscale view on the superconductivity of electro-reduced STO regarding the doped dislocations as metallic filaments, whose cores have similar properties to Ti_2_O_3_ or TiO [69,72,73]. These dislocation-based filaments are connected among each other due to the invariance of the Burgers vector [99] and thus form a metallic network in the dielectric insulating STO matrix. Although low Ti oxides are superconducting per se, their presence in the core of the dislocations alone cannot explain the superconductivity enhancement in dislocation-rich STO. The radius of the dislocation cores equals 1–2 nm [31] and is much smaller than the correlation length of Cooper pairs expected in bulk Ti/TiO materials according to the Bardeen–Cooper–Schrieffer (BCS) theory [83], and smaller than the coherence length determined for our electro-reduced crystal by the upper critical field (40–50 nm).

We conclude that superconductivity is related to a cooperative phenomenon involving the metallic cores of dislocations and the polar regions between them. As schematically illustrated in Figure 13d, the d electrons in the metallic dislocations are coupled by the polar properties of the STO matrix. In the polar region between the nano-filaments, a static spontaneous polarization (green arrows in Figure 13d) and a dynamic polarization (golden spheres in Figure 13d) are present. Our DFT simulations indicate that the spontaneous polarization in the STO matrix coexists with metallic filaments. However, the electrons provided by the filaments lightly dope the surrounding matrix, initializing interband interactions. The possibility of generating a dynamic polarization in nano-regions at such a doping level was confirmed by lattice dynamic calculations. In other words, the proximity of the metallic filaments does not eliminate the polar character of the STO matrix, but provides the charge carriers necessary for superconductivity. Accordingly, the interaction of the polarization with the d^1^ electronic states in different filaments of a bundle constitutes the glue of the Cooper pairs and thus leads to local superconductivity. The determined coherence length perfectly correlates with the average radius of the bundles formed by electro-degradation. Self-doped STO is highly inhomogeneous with coexisting charge-rich and charge-poor regions, i.e., a material in which metallicity and polar properties are intimately entangled. Although the details of the coupling mechanism of this unconventional superconductor remain to be discovered in the future, we have demonstrated that the two prerequisites for superconductivity, namely an appropriate charge carrier density and a local polarization, are present within the bundles, thus promising a route for the artificial local embedding of superconductivity in insulating polar materials.

## Figures and Tables

**Table 1:** Effusion of oxygen from an STO substrate during reduction under UHV conditions.

Temperature (°C)	Amount of Effused Oxygen from an STO Crystal with Size 1 × 1 × 0.05 cm^3^
600	1.3 × 10^12^ *
700	2.1 × 10^12^ *
800	1.6 × 10^12^
900	5.5 × 10^12^
1000	5.3 × 10^12^

* For 600 and 700 °C, a maximum rate of calcination of SrCO_3_ was observed by operando XPS (O1s and C1s). This process is limited to the SrO-terminated surface with chemisorbed CO_2_ [31], leading to the desorption of CO_2_. At this high temperature, the sources of emitted oxygen are not only the core of dislocations, but also the decomposition of CO_2_→CO + ½O_2_.

**Table 2:** Comparison of the total amount of effused oxygen of an STO sample (cf. Table 1) with the estimated local oxygen concentration close to preferentially reduced dislocations.

Temperature (°C)	Total Effused Oxygen	Increase in Oxygen Non-Stoichiometry in the Dislocation Network *	Total Oxygen Non-Stoichiometry in the Network
800	1.6 × 10^12^	1.9 × 10^19^/cm^3^	1.9 × 10^19^/cm^3^
900	5.5 × 10^12^	6.6 × 10^19^/cm^3^	8.5 × 10^19^/cm^3^
1000	5.3 × 10^12^	6.4 × 10^19^/cm^3^	1.5 × 10^20^/cm^3^

* The values for 600 and 700° were not considered due to the limitations discussed in Table 1.

## Data Availability

The original contributions presented in this study are included in the article/Supplementary Materials. Further inquiries can be directed to the corresponding author.

## Supplementary Materials

The following supporting information can be downloaded at: https://www.mdpi.com/article/10.3390/nano14231944/s1, Figure S1. Progression of the electro-reduction of SrTiO_3_ (100) crystals (T = 350 °C, vacuum) for two polarization modes. Figure S2. The temperature-dependence of the electrical resistance for different regions of the electro-reduced crystal. Figure S3. Temperature-dependence of the resistance of SrTiO_3_ crystals during heating in air and subsequent cooling under vacuum conditions. Figure S4. Microscopic view on the electro-reduction of STO. Figure S5. Progression of the thermal reduction of an SrTiO_3_ crystal at 800 °C in a vacuum for different regions of the samples. Refs. [30,55,64,66,82,100,101,102,103,104] are cited in the supplementary materials.
