# Supplementary material for: Transition to Metallic and Superconducting States Induced by Thermal or Electrical Deoxidation of the Dislocation Network in the Surface Region of SrTiO3
Source: Nanomaterials (Basel). 2024 Dec 4;14(23):1944. doi: 10.3390/nano14231944 (PMC11643870; doi:10.3390/nano14231944)
Supplement: Supplementary file 1 [file nanomaterials-14-01944-s001.zip › nanomaterials-3332653-supplementary.pdf]

Supplementary information to

Transition to metallic and superconducting states induced by thermal or electrical deoxidation of the dislocation network in the surface region of $SrTiO_3$

K. Szot, C. Rodenbücher, K. Rogacki, G. Bihlmayer, W. Speier, K. Roleder, F. Krok, H. Keller, A. Simon and A. Bussmann-Holder

Electro-reduction experiment

The typical progression of the electro-reduction at a constant polarization voltage as a function of time is displayed in Figure S1a. The evolution of the resistance in stepwise constant current mode is shown in Figure S1b. In this mode, the current was increased in steps from 1 nA to 1 mA. As a criterion for switching to the next current step, a threshold voltage of 1 V was selected. Although the resistance decreased nearly continuously in constant voltage mode, the decrease in the resistance when using the stepwise constant current mode reached the maximum progression when the current was switched to 0.1, 1, 10 mA. It can be observed that in a relatively short time period, the resistance in all regions near the cathode, in the bulk, and near the anode was reduced by orders of magnitude. It should be noted that the time-dependent resistance changes during electro-reduction for different samples (e.g., from other suppliers or different batches) can exhibit fully dissimilar behavior, despite the same experimental parameters being present. This “undefined behavior” relates to the influence of different growth conditions and sample preparation (e.g., polishing methods) on the properties of the crystals and, in particular, on the electrical transport phenomena (for details, see Szot et al. [1]). In order to analyze the ex-corporation of oxygen during electro-reduction, we simultaneously measured the effusion of oxygen using a quadrupole mass spectrometer [1]. The mass spectrometric analysis revealed that during electro-reduction in stepwise constant current mode, an outflow of oxygen from the sample of the order of $10^{15}/cm^3$ was present. The oxygen outflow reached its maximum in the first stage of electro-reduction when the current was progressively increased. Nevertheless, after a few minutes, the oxygen concentration in the chamber decreased to the level it had been prior to the commencement of the electro-reduction. The extremely low oxygen concentration of only $10^{15}/cm^3$ that accompanied the electro-reduction process was calculated assuming that the oxygen vacancies were homogeneously generated in bulk. However, the electro-reduction of $SrTiO_3$ crystals was found to be fully inhomogeneous and restricted only to the dislocation cores arranged in a hierarchical network in the surface region. For such a selective de-oxidation generating a three-dimensional network of filaments, the actual local defect concentration in the reduced dislocation core was dramatically higher and can be assumed to be in the range of $10^{20\text{-}21}/cm^3$. Although electro-reduction can result in a decrease in the sample’s original resistivity by 6–7 orders of magnitude (cf. Figure S1), this large drop in resistivity does not mean that the entire sample was converted into a metallic state. Therefore, the resistance dependence as a function of the temperature was analyzed after electro-reduction, as is shown in Figure S2. Two different electro-reduced crystals were compared. Figure S2a reveals a case, in which conversion to the metallic state

upon electro-reduction in step-wise constant current mode (I = 1 nA - 10 mA, T = 320 °C, vacuum) only occurred in the bulk and the near-cathode region, as identified by the increasing resistance with temperature. Still, the near-anode region exhibited a behavior typical for a semiconductor. The investigation of the second sample, which was electro-reduced in constant voltage mode (U = 300 V, I_{comp} = 10 mA, T = 300 °C, vacuum) revealed metallicity in all regions (Figure S2b). This indicates that metallic filaments formed in the entire surface layer, guaranteeing that sufficient current can flow through the sample, even at low temperatures. Hence, this sample was selected for the superconductivity investigation.

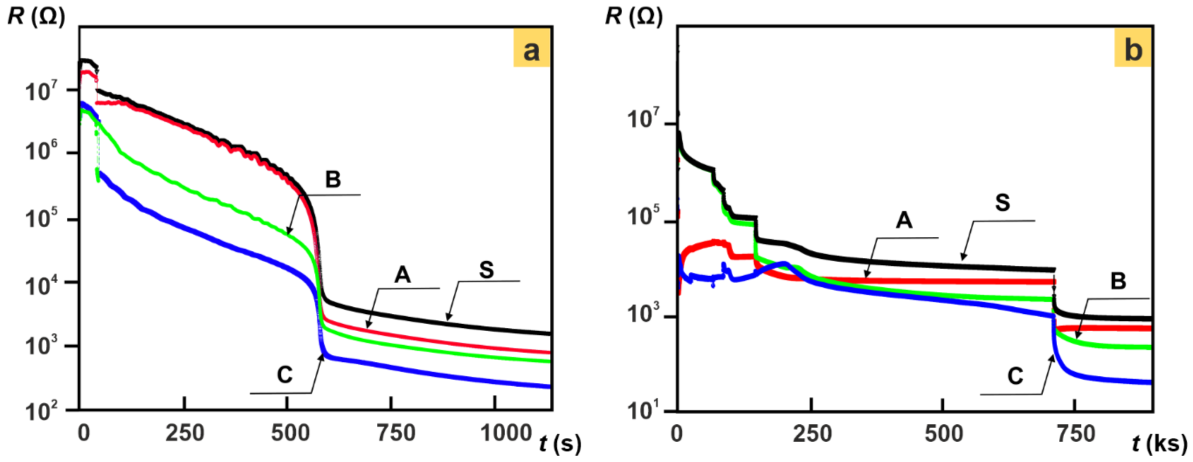

Figure S1. Progression of the electro-reduction of $SrTiO_3$ (100) crystals (T = 350 °C, vacuum) for two polarization modes. a) Constant voltage (200 V); b) stepwise constant current: 0↔11s:10^{-9}A, 11↔48s:10^{-8}A, 48↔160s:10^{-7}A, 160↔6928s:10^{-6}A, 6928↔8905s:10^{-5}A, 8905↔14820s:10^{-4}A, 14820↔71258s:10^{-3}A, and 71258↔end:10^{-2}A.
The letters above the curves are abbreviations for: S-whole sample, B-bulk, A-region close to the anode, and C- region close to the cathode.

Despite the clear indication that the low-temperature (350 °C) electro-reduction under vacuum relates to the electrically-induced de-oxidation of the core of the filaments, it may be asked whether the thermal treatment by Joule heating under vacuum conditions can also contribute to the decrease in macroscopic resistance. Through measurement, we determined that during electro-reduction with 10 mA, the increase in the sample's resistance due to Joule heating was well below 100 ºC [2]. Therefore, we checked the influence of thermal conditions on the potential self-doping of our crystals. A pristine crystal was heated in air to 380 °C. Then, the chamber was evacuated and the temperature was maintained for four hours before the sample was cooled to room temperature. Figure S3 shows that the resistance measured versus the temperature exhibited nearly identical semiconducting behavior during heating in air and cooling in a vacuum proving that no significant thermal reduction occurred at this temperature.

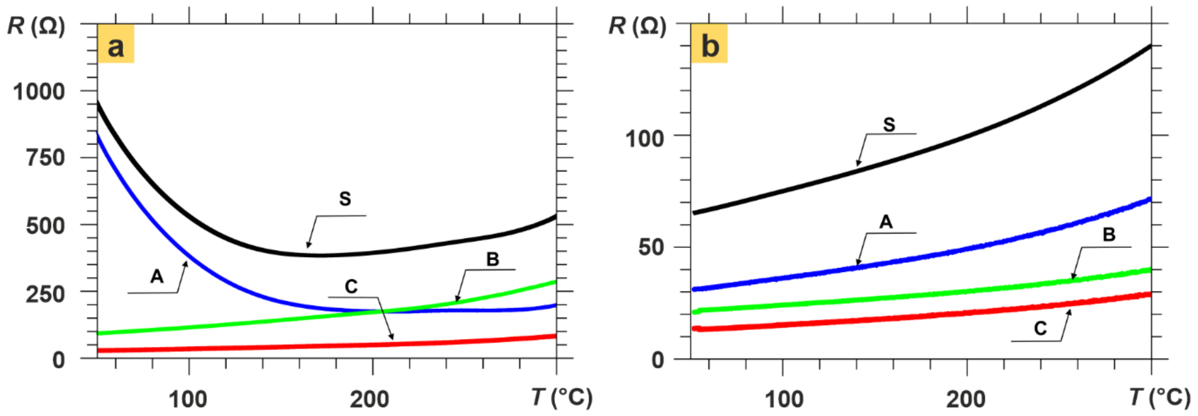

Figure S2. The temperature-dependence of the electrical resistance for different regions of the electro-reduced crystal. a) Metallic transformation of the region close to the anode only and **b**) full transformation of the entire sample into a metallic state (the notation S, B, A, C is the same as in Figure S1).

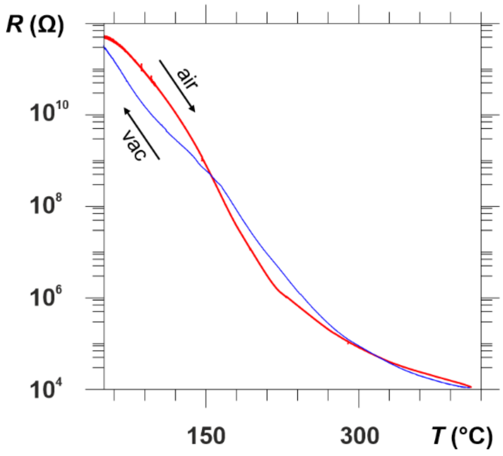

Figure S3. Temperature-dependence of the resistance of $SrTiO_3$ crystals during heating in air and subsequent cooling under vacuum conditions. At the maximal temperature of approximately 400 ºC, the sample was annealed for 4 h under vacuum conditions.

LC-AFM investigation of the electrical conductivity on the nanoscale

Following electro-reduction, the samples were cooled to room temperature under vacuum conditions, but their transfer from the preparation chamber to the cryostat was undertaken ex-situ. Although this transfer was performed at room temperature, the freshly-prepared sample was exposed to the ambient atmosphere. To ensure comparability, the LC-AFM analysis was also performed after exposing the sample to ambient conditions (see main text). For the LC-AFM mapping of the electrical conductivity on the crystals' cross-sections (Figure S4), the samples were cleaved in situ. In order to prevent post-oxidation of the freshly-cleaved surfaces, the cleavage process, and finally, the measurement, was carried out under vacuum conditions with the partial pressure of the oxygen reduced to 10^{-15}–10^{-17} mbar by the addition of H_2 to the chamber at a total pressure of 10^{-6} mbar.

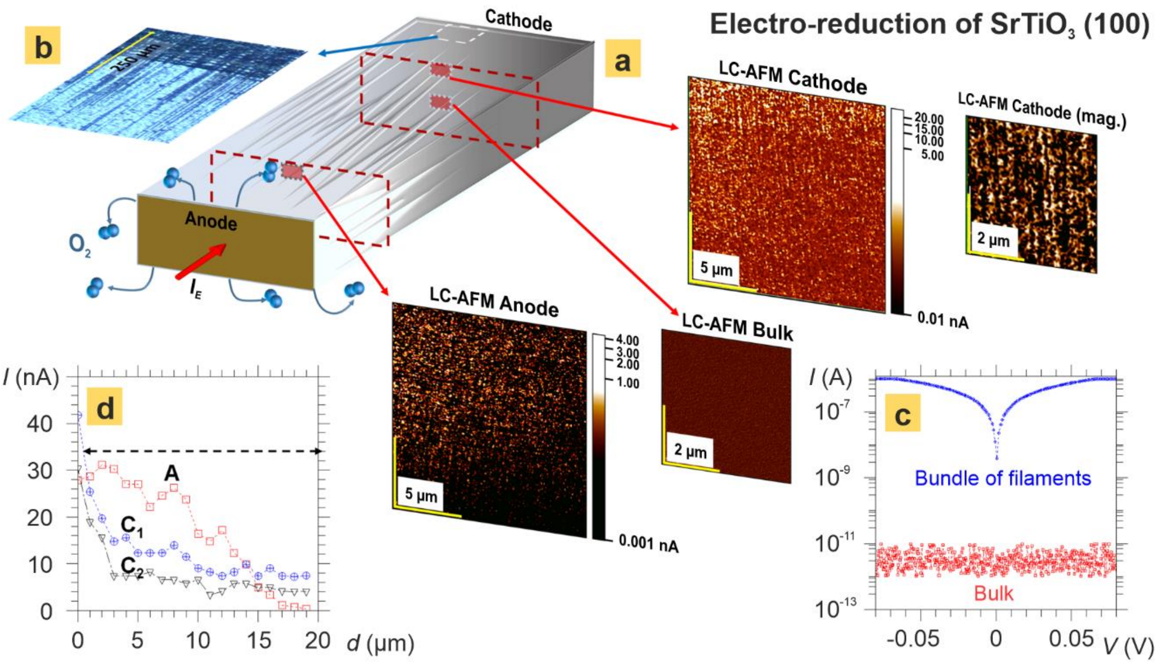

Figure S4. Microscopic view on the electro-reduction of STO. a) Schematic view of the experimental situation for electro-reduction of STO with cross-sectional LC-AFM maps obtained after cleaving the crystal in the anode and cathode regions (with magnification, mag.); b) micrograph of the distribution of dislocations in the cathode region revealed by the etch-pits technique; c) I/V measurements obtained by LC-AFM on a bundle of filaments and in the bulk region; d) average current as a function of the distance to the surface as calculated from the LC-AFM maps close to the anode (A) and in two positions close to the cathode (C1, C2).

In order to analyze the morphology of the network of conducting filaments induced by electro-reduction, cross sections of the crystals were analyzed by LC-AFM. Figure S4 illustrates the positions, where the cross sectioning was performed. The maps obtained in the cathode region after cleaving the crystal (Figure S4a) and the optical micrograph of the exits of dislocations as revealed by etch-pits technique (Figure S4b) reveal the inhomogeneous distribution of filamentary structures following electro-reduction. Close to the cathode, a higher concentration of electro-reduced filaments was present than in the vicinity of the anode due to the electro-migration of the oxygen ions in the electrical field gradient. The LC-AFM maps exhibit a hierarchical character of the conducting filaments in the surface region, similar to the filaments in the surface region of thermally-reduced crystals, thus supporting the idea that dislocations predefine the shape of the filamentary network. In all regions of the sample, well-conducting filaments only existed in the last few tens of micrometers of the surface region, whereas the cross-sectional LC-AFM maps of the bulk do not show conducting filaments, and so evidence that the bulk of the crystal remains insulating. The lack of electrical conductivity can also be identified for the matrix region between the filaments if the distance between the filaments is larger than a few nanometers. This is indicated by I/V measurements obtained by contacting either a filament or matrix with the LC-AFM tip (Figure S4c). The results for the filaments exhibit a linear current–voltage dependence (which is curved in the logarithmic representation), whereas the current in the bulk region was below the detection limit. To illustrate the depth dependence of the conductivity, the mean current flow at the cathode (C1 and C2) and anode (A) is presented in Figure S4d. Each point of the curves was

calculated by integrating the current of an "LC-AFM strip" with an area of 5 × 1 μm^2, starting from the surface to the strip at a distance of 19 µm below the surface. Close to the surface, the averaged current was highest, and then dropped significantly within a few micrometers, revealing the "skin-like character" of the electric transport, with the electro-formed surface region carrying the lion's share of the total macroscopic current. Using a grain analysis, the dimension of the conducting spots in the LC-AFM maps was determined to be approximately 45 nm.

A strong agglomeration of filaments into orthogonal bands in the [100] direction was present near the cathode as can be seen as stripe-like structures, both in the etch-pits and the LC-AFM maps of Figure S4. The LC-AFM maps indicate that the filaments do not occur as single filaments isolated from each other, but form filament bundles. In fact, the LC-AFM maps indicate that the filaments do not occur as single filaments isolated from each other but form filament bundles. This bundling effect was also observed in thermally-reduced crystals [3], but is much more pronounced for electro-reduced crystals.

Self-doping of $SrTiO_3$ crystals caused by thermal reduction

Thus far, superconductivity in stoichiometric $SrTiO_3$ has only been explored for thermally-reduced crystals. Hence, for the analysis of the superconducting properties of electro-reduced crystals, we were obliged, *per se*, to compare the properties of electro-reduced crystals with thermally-prepared ones. To avoid an influence of the sample preparation process due, e.g., to different growth parameters, polishing methods and concentrations of impurities on the progression of both the reduction and electro-reduction processes, the experiments were performed on samples prepared from the same crystal.

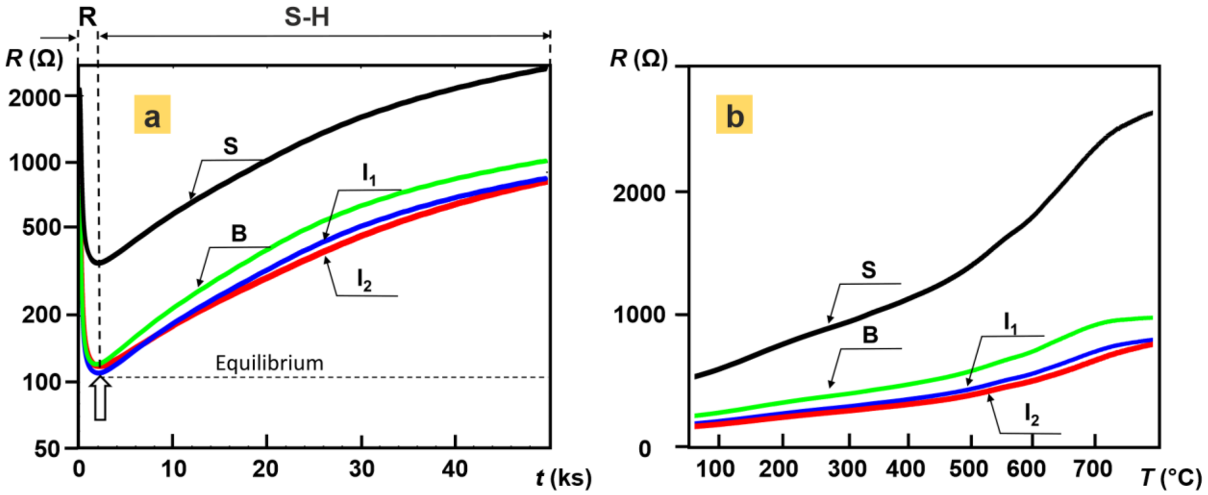

Figure S5. Progression of the thermal reduction of an $SrTiO_3$ crystal at 800 °C in a vacuum for different regions of the samples. a) Resistance as a function of reduction time (S-whole sample, B-bulk, and I_1-I_2-Interfaces close to electrodes). The position of the white arrow marks the optimal reduction state (R) induced in the crystal. The prolonged reduction does not allow for the reaching of the equilibrium of the defects, but leads to the self-healing process (S-H); in the depicted process, the resistance increases by a factor of > 5 (after a long reduction time) relative to the maximum reduction state reached after only a few minutes [4]. **b**) thermal dependence of the resistivity of a thermally reduced

crystal revealing that the reduction process results in the conversion of all regions in the crystals into the metallic state.

For the thermally-induced self-doping of the crystal, we chose an annealing temperature of about 800 ºC and a pressure of 10^{-7} mbar. In contrast to an *uncontrolled* reduction process, as would be caused by placing the crystals in the hot area of the furnace in the vacuum chamber and waiting until equilibrium was reached, we analyzed the progression of thermal reduction by monitoring the resistance as a function of the reduction time (Figure S5-left) as described by Rodenbücher et al. [5]. It can be seen that the resistance initially decreases and then increases again as a result of self-healing [4]. The presence of the minimum on the curve $R(t)_{T\text{const},p\text{const}}$ (marked with an arrow) clearly shows that it is necessary to stop the reduction after a certain time in order to obtain the maximum reduction or maximum concentration of self-doped carriers. It is surprising that this thermal reduction, which transformed the sample into a metallic state (see Figure S5, right) and finally into a superconducting crysta), only results in $10^{14}/cm^3$ oxygen atoms leaving the entire sample [1,4].

Hence, the macroscopically-determined increase in resistivity cannot be interpreted as the effect of self-doping of the entire crystal, but is an effect of the selective removal of oxygen from the dislocation network in the surface region [1,6,7]. In this regard, thermal reduction and electro-reduction are similar, as both processes occur, in principle, in the hierarchical network in dislocations. Of course, based on the point defect chemistry for the reduced ternary oxides at low oxygen activity (here, the vacuum) levels, it can be assumed that “classical doping” of the matrix should occur. It is assumed that the two-fold positive charge of oxygen vacancies is compensated by electrons under reducing conditions, thus turning the matrix into an n-type semiconductor. As the LC-AFM measurement of the electrical conductivity of the matrix (e.g., between filaments and in the bulk [8,9]) in thermally-reduced crystals exhibits no decrease in resistivity, we can conclude that such a potential homogeneous doping with electrons is not relevant for the nano- and macroscopic transition to the metallic state. The matrix of thermally-reduced crystals is just as isolating as in the case of stoichiometric crystals [10]. In-situ 4-tip STM investigations [7] show that the thermal reduction that occurs in the surface layer only provides a further proof of the inhomogeneity of the thermal reduction.

1. Szot, K. *et al.* Influence of Dislocations in Transition Metal Oxides on Selected Physical and Chemical Properties. *Crystals* **8**, 241 (2018).
2. Rodenbücher, C. *et al.* Current channeling along extended defects during electroreduction of $SrTiO_3$. *Scientific Reports* **9**, 2502 (2019).
3. Wrana, D., Rodenbücher, C., Bełza, W., Szot, K. & Krok, F. In situ study of redox processes on the surface of $SrTiO_3$ single crystals. *Applied Surface Science* **432**, 46–52 (2018).
4. Szot, K., Speier, W., Carius, R., Zastrow, U. & Beyer, W. Localized Metallic Conductivity and Self-Healing during Thermal Reduction of $SrTiO_3$. *Physical Review Letters* **88**, 075508 (2002).
5. Rodenbücher, C., Korte, C., Schmitz-Kempen, T., Bette, S. & Szot, K. A physical method for investigating defect chemistry in solid metal oxides. *APL Materials* **9**, 011106 (2021).

6. Rodenbücher, C., Wojtyniak, M. & Szot, K. Conductive AFM for Nanoscale Analysis of High-k Dielectric Metal Oxides. in *Electrical Atomic Force Microscopy for Nanoelectronics* (ed. Celano, U.) 29–70 (Springer International Publishing, 2019). doi:10.1007/978-3-030-15612-1_2.
7. Leis, A. *et al.* In-situ four-tip STM investigation of the transition from 2D to 3D charge transport in $SrTiO_3$. *Scientific Reports* **9**, 2476 (2019).
8. Szot, K., Speier, W., Bihlmayer, G. & Waser, R. Switching the electrical resistance of individual dislocations in single-crystalline $SrTiO_3$. *Nature Materials* **5**, 312–320 (2006).
9. Waser, R., Dittmann, R., Staikov, G. & Szot, K. Redox-Based Resistive Switching Memories - Nanoionic Mechanisms, Prospects, and Challenges. *Advanced Materials* **21**, 2632–2663 (2009).
10. Rodenbücher, C. *et al.* The Electronic Properties of Extended Defects in $SrTiO_3$—A Case Study of a Real Bicrystal Boundary. *Crystals* **10**, 665 (2020).
